# Data and its (dis)contents: A survey of dataset development and use in machine learning research

**DOI:** 10.1016/j.patter.2021.100336

**Published:** 2021-11-12

**Authors:** Amandalynne Paullada, Inioluwa Deborah Raji, Emily M. Bender, Emily Denton, Alex Hanna

**Affiliations:** 1 Department of Linguistics, University of Washington, Seattle, WA, USA; 2 Google Research, New York, NY, USA; 3 Mozilla Foundation, Mountain View, CA, USA; 4 Google Research, San Francisco, CA, USA

**Keywords:** datasets machine learning

## Abstract

In this work, we survey a breadth of literature that has revealed the limitations of predominant practices for dataset collection and use in the field of machine learning. We cover studies that critically review the design and development of datasets with a focus on negative societal impacts and poor outcomes for system performance. We also cover approaches to filtering and augmenting data and modeling techniques aimed at mitigating the impact of bias in datasets. Finally, we discuss works that have studied data practices, cultures, and disciplinary norms and discuss implications for the legal, ethical, and functional challenges the field continues to face. Based on these findings, we advocate for the use of both qualitative and quantitative approaches to more carefully document and analyze datasets during the creation and usage phases.

## The bigger picture

Datasets form the basis for training, evaluating, and benchmarking machine learning models and have played a foundational role in the advancement of the field. Furthermore, the ways in which we collect, construct, and share these datasets inform the kinds of problems the field pursues and the methods explored in algorithm development. In this work, we survey recent issues pertaining to data in machine learning research, focusing primarily on work in computer vision and natural language processing. We summarize concerns relating to the design, collection, maintenance, distribution, and use of machine learning datasets as well as broader disciplinary norms and cultures that pervade the field. We advocate a turn in the culture toward more careful practices of development, maintenance, and distribution of datasets that are attentive to limitations and societal impact while respecting the intellectual property and privacy rights of data creators and data subjects.

## Teaser

Datasets have become a critical component in the advancement of machine learning research. The ways in which such datasets are collected, constructed, and shared play a significant role in shaping the quality and impact of this research. We conduct a survey of the literature on concerns relating to the design, collection, maintenance, and distribution of machine learning datasets, as well as broader disciplinary norms and cultures that pervade the field.

## Introduction

The importance of datasets for machine learning research cannot be overstated. Datasets have been seen as the limiting factor for algorithmic development and scientific progress,^1^^,^^2^ and a select few benchmark datasets, such as the ImageNet benchmark for visual object recognition^3^ and the GLUE benchmark for English textual understanding,^4^ have been the foundation for some of the most significant developments in the field. Benchmark datasets have also played a critical role in orienting the goals, values, and research agendas of the machine learning community.^5^ In recent years, machine learning systems have been reported to achieve “super-human” performance when evaluated on such benchmark datasets. However, recent work from a variety of perspectives has surfaced not only the shortcomings of some machine learning datasets as meaningful tests of human-like reasoning ability, but also the troubling realities of the societal impact of how these datasets are developed and used. Together, these insights reveal how this apparent progress may rest on faulty foundations.

As the machine learning field turned to approaches with larger data requirements in the last decade, the sort of skilled and methodical annotation applied in dataset collection practices in earlier eras was spurned as “slow and expensive to acquire,” and a turn toward unfettered collection of increasingly large amounts of data from the web, alongside increased reliance on crowdworkers, was seen as a boon to machine learning.^1^^,^^3^^,^^6^^,^^7^ The enormous scale of such datasets has been mythologized as beneficial to the perceived generality of trained systems,^7^ but they continue to be impacted by the limitations and biases that impact all datasets.^8^ In particular, prevailing data practices tend to abstract away the human labor, subjective judgments and biases, and contingent contexts involved in dataset production. However, such details are important for assessing whether and how a dataset might be useful for a particular application, for enabling more rigorous error analysis, and for acknowledging the significant difficulty required in constructing useful datasets.

The machine learning field has placed large-scale datasets at the center of model development and evaluation. As systems trained in this way are deployed in real-world contexts that affect the lives and livelihoods of real people, it is essential that researchers, advocacy groups, and the public at large understand both the contents of the datasets and how they affect system performance. In particular, as the field has focused on benchmarks as the primary tool for both measuring and driving research progress,^9^ understanding what these benchmarks measure (and how well) becomes increasingly urgent.

**Figure 1:**
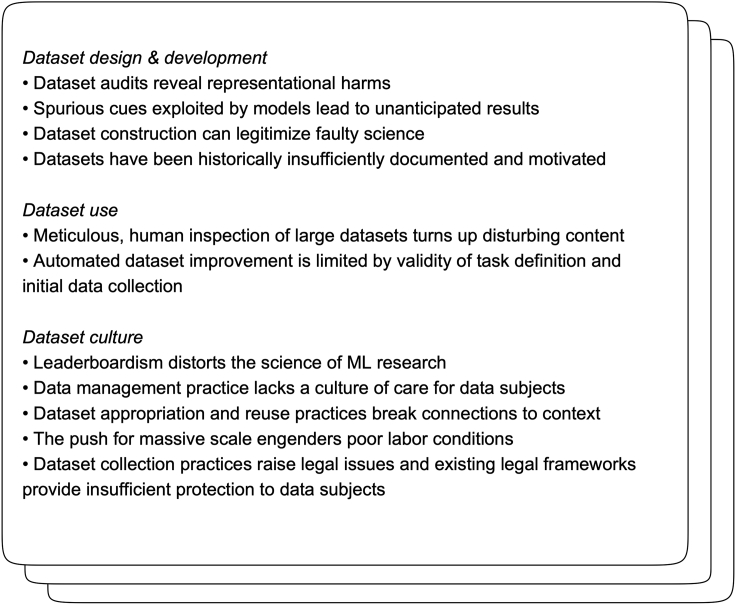
Key takeaways from a survey of perspectives on the challenges posed by recent trends in dataset use in machine learning

We thus conduct a survey of the literature of recent issues pertaining to data in machine learning research, with a particular focus on work in computer vision and natural language processing (NLP). We structure our survey around three themes. The first, Dataset design and development, deals with studies that critically review the design of the datasets used as benchmarks. This includes studies that audit existing datasets for bias, those that examine existing datasets for spurious correlations which make the benchmarks gameable, those that critically analyze the framing of tasks, and work promoting better data collection and documentation practices. Next, in Dataset in(tro)spection, we review approaches to exploring and improving these aspects of datasets. In looking at approaches to filtering and augmenting data and modeling techniques aimed at mitigating the impact of bias in datasets, we see further critiques of the current state of datasets. However, we find that these approaches do not fully address the broader issues with data use. Finally, in Dataset culture, we survey work on dataset practices as a whole, including critiques of their use as performance targets, perspectives on data management and reuse, research into the precarious labor conditions that underpin much of dataset production, and papers raising legal issues pertaining to data collection and distribution. The key findings of the sections which form the body of the paper are summarized in Figure 1.

## Definitions

We follow Schlangen^9^ in distinguishing between *benchmarks*, *tasks*, *capabilities*, and *datasets*. While his work focused on NLP, we broaden these definitions to include aspects of other machine learning applications. In this context, a *task* is constituted of an input space and output space and an expected mapping between them. Schlangen notes that there are typically both *intensional* and *extensional* definitions of tasks. An intensional definition describes the relationship between input and output (e.g., the output in automatic speech recognition is a transcription of the audio signal in the input), where an extensional definition is simply the set of input-output pairs in the dataset. Thus tasks are exemplified by *datasets*, i.e., sets of input-output pairs that conform, if valid, to the intensional definition of the task. Tasks can be of interest for two (not mutually exclusive) reasons: either they map directly to a use case (e.g., automatic transcription of audio data) or they illustrate cognitive *capabilities*, typical of humans, that we are attempting to program into machines. In the former case, a task is suitable as a *benchmark* (for comparing competing systems to each other) if the task is well-aligned with its real-world use case and the dataset is sufficiently representative of the data the systems would encounter in production. In the latter case, establishing the value of the task as a benchmark is more involved: as Schlangen argues, success on the task has to be shown to rely on having some set of capabilities that are definable outside of the task itself and transferable to other tasks.

In referring to dataset exemplars that pair instances (input) and labels (output), we follow a convention from machine learning of referring to the latter as *target labels*, which are those labels that are used as the learning target, and which have typically been produced by human annotators or, in some cases, automated labeling heuristics. These are also often referred to in the literature as “gold standard” or “ground truth” labels, but we wish to emphasize their role as training targets that are neither objective nor necessarily representative of reality.

## Dataset design and development

“Raw data is both an oxymoron and a bad idea; to the contrary, data should be cooked with care.”—Geoffrey Bowker (*Memory Practices in the Sciences*)^10^

In this section, we review papers that explore issues with the contents of datasets that arise due to the manner in which they were collected, the assumptions guiding the dataset construction process, and the set of questions guiding their development.

### Representational harms in datasets

In recent years there has been growing concern regarding the degree and manner of representation of different sociodemographic groups within prominent machine learning datasets, constituting what Kate Crawford has called *representational harms.*^11^ For example, a glaring under-representation of darker-skinned subjects, compared with lighter-skinned subjects, has been identified within prominent facial analysis datasets^6^^,^^12^ and in image datasets used to train self-driving cars to detect pedestrians.^13^ Meanwhile, the images in object recognition datasets have been overwhelmingly sourced from Western countries.^14^ Zhao et al.^15^ found a stark under-representation of female pronouns in the commonly used OntoNotes dataset for English coreference resolution; similarly, Lennon^16^ found that feminine-coded names were vastly underrepresented in the CoNLL-2003 dataset used for named entity recognition. While the under-representation of marginalized groups in datasets has been met with calls for “inclusion,” Hoffmann^17^ provides a case for skepticism of this narrative, as it has the potential to merely uphold the very sort of power hierarchy that engenders such under-representation in the first place.

Stereotype-aligned correlations have also been identified in both computer vision and NLP datasets. For example, word co-occurrences in NLP datasets frequently reflect social biases and stereotypes relating to race, gender, (dis)ability, and more^18^^,^^19^ and correlations between gender and activities depicted in computer vision datasets have been shown to reflect common gender stereotypes.^20^, ^21^, ^22^ Dixon et al.^23^ found that a dataset for toxicity classification contained a disproportionate association between words describing queer identities and text labeled as “toxic,” while Park et al.^24^ found evidence of gender bias against women in similar datasets. Such disparities in representation stem, in part, from the fact that particular, non-neutral viewpoints are routinely yet implicitly invoked in the design of tasks and labeling heuristics. For example, a survey of literature on computer vision systems for detecting pornography found that the task is largely framed around detecting the features of thin, nude, female-presenting bodies with little body hair, largely to the exclusion of other kinds of bodies—thereby implicitly assuming a relatively narrow and conservative view of pornography that happens to align with a straight male gaze.^25^

In an examination of the person categories within the ImageNet dataset,^3^ Crawford and Paglen^26^ uncovered millions of images of people that had been labeled with offensive categories, including racial slurs and derogatory phrases. In a similar vein, Birhane and Prabhu^27^ examined a broader swath of image classification datasets that were constructed using the same categorical schema as ImageNet, finding a range of harmful and problematic representations, including non-consensual and pornographic imagery of women. In response to the work of Crawford and Paglen,^26^ a large portion of the ImageNet dataset has been removed.^28^ Similarly, Birhane and Prabhu's examination^27^ prompted the complete removal of the TinyImages dataset.^29^

### Spurious cues exploited by machine learning models

While deep learning models have seemed to achieve remarkable performance on challenging tasks in artificial intelligence, recent work has illustrated how these performance gains may be due largely to “cheap tricks” (to borrow a term from Levesque^30^) rather than human-like reasoning *capabilities*, as defined in Definitions. Geirhos et al.^31^ illustrate how the performance of deep neural networks can rely on *shortcuts*, or decision rules that do not extrapolate well to out-of-distribution data and are often based on incidental associations. Oftentimes, these shortcuts arise due to artifacts in datasets that allow models to overfit to training data and to rely on nonsensical heuristics to “solve” the task—for example, detecting the presence of pneumonia in chest X-ray scans based on hospital-specific tokens that appear in the images.^31^ That is, despite high predictive performance, models are not performing the task according to its *intensional* description, and thus the datasets may not be exemplary of reasoning *capabilities*. Recent work has revealed the presence of shortcuts in commonly used datasets that had been conceived of as proving grounds for particular competencies, such as reading comprehension and other “language understanding” capabilities. Experiments that illuminate such data artifacts, or “dataset ablations” as Heinzerling^32^ calls them, involve simple or nonsensical baselines, such as training models on incomplete inputs and comparing performance to models trained on full inputs. Much recent work in NLP has revealed how these simple baselines are competitive, and that models trained on incomplete inputs for argument reasoning, natural language inference, fact verification, and reading comprehension—i.e., tasks restructured in such a way that there should be no information about the correct output in the input—perform quite well.^33^, ^34^, ^35^, ^36^, ^37^ (Storks et al.^38^ and Schlegel et al.^39^ provide more comprehensive reviews of datasets and dataset ablations for natural language inference.) In many cases, this work has revealed how an over-representation of simple linguistic patterns (such as negation or presence of certain words) in dataset instances belonging to one label class can serve as a spurious signal for models to pick up on. Many of these issues result from the assumptions made in task design and in the under-specification of instructions given to human data labelers, and can thus can be addressed by rethinking the format that dataset collection takes. In light of this, recent work has proposed approaches to pre-empting spurious correlations by designing annotation frameworks that better leverage human “common sense”^40^ and more critical approaches to dataset creation and use for tasks such as reading comprehension.^41^

### How do datasets legitimize certain problems or goals?

As the previous sections have laid out, the mapping between inputs and target labels contained in datasets is not always a meaningful one, and the ways in which data are collected and tasks are structured can lead models to rely on faulty heuristics for making predictions. The problems this raises are not limited to misleading conclusions based on benchmarking studies: when machine learning models can leverage spurious cues to make predictions well enough to beat a baseline on the test data, the resulting systems can appear to legitimize spurious tasks that do not map to real-world capabilities. More formally, there are some tasks that can be described *intensionally* but for which there is no possibility of a sufficient *extensional* realization, often because the underlying theory for the task is unsound.

Decisions about what data to collect in the first place and the problematization that guides data collection lead to the creation of datasets that formulate pseudoscientific tasks. For example, several studies in recent years that attempt to predict attributes such as sexuality and other fluid, subjective personal traits from photos of human faces presuppose that these predictions are possible and worthwhile to make. However, these datasets, like those discussed above, enable a reliance on meaningless shortcuts. These in turn support the apparent “learnability” of the personal traits in question. An audit by Agüera y Arcas et al.^42^ found that a model trained to predict sexual orientation from images of faces harvested from online dating profiles was actually learning to spot stereotypical choices in grooming and self-expression, which are by no means universal, while Gelman et al. discuss how such a study strips away context and implies the existence of an “essential homosexual nature”.^43^ The task rests on a pseudoscientific essentialism of human traits. Another example, from NLP, is GermEval 2020 Task 1,^44^ which asked systems to reproduce a ranking of students by IQ scores and grades using only German short answer texts produced by the students as input. By setting up this task as feasible (for machine models or otherwise), the task organizers suggested that short answer texts contain sufficient information to “predict” IQ scores and furthermore that IQ scores are a valid and relevant thing to measure about a person.^45^ Jacobsen et al.^46^ point out that shortcuts in deep learning, as described in Section 3.2, make ethically dubious questions seem answerable, and advise, “[W]hen assessing whether a task is solvable, we first need to ask: should it be solved? And if so, should it be solved by AI?” Not only are these task formulations problematic, but, as we describe in Section 5.3, once sensitive data has been collected, it can be misused.

### Collection, annotation, and documentation practices

A host of concerns regarding the practices of dataset collection, annotation, and documentation have been raised within recent years. In combination, these concerns reflect what Jo and Gebru^47^ describe as a *laissez-faire* attitude regarding dataset development and the pervasive undervaluation of data work.^48^ Rather than collecting and curating datasets with care and intentionality—as is more typical in other data-centric disciplines—machine learning practitioners often adopt an approach where anything goes. As one data scientist put it, “if it is available to us, we ingest it.”^49^

The common practices of scraping data from internet search engines, social media platforms, and other publicly available online sources faced significant backlash in recent years. For example, facial analysis datasets have received push-back due to the inclusion of personal Flickr photos without data subjects’ knowledge.^50^ In many instances, the legality of the data usage has come into question, as we discuss further in Legal perspectives.

Dataset annotation practices have also come under great scrutiny in recent years. Much of this has focused on how subjective values, judgments, and biases of annotators contribute to undesirable or unintended dataset bias.^22^^,^^51^, ^52^, ^53^, ^54^ More generally, several researchers have identified a widespread failure to recognize annotation work as *interpretive work*, which in turn can result in a conflation of *target* labels in a collected dataset and *real-world* objects, for which there may be no single ground truth label.^55^^,^^56^ As discussed further in Labor, data annotation tasks are often mediated through crowdwork platforms such as Amazon Mechanical Turk (AMT). These platforms, by design, position annotators as interchangeable workers, rather than individuals who bring to bear their own subjective experiences and interpretations to the task. Divergences in judgments across different annotator pools,^57^ as well as between AMT annotators and other communities,^58^ have been empirically explored.

Recent work by Tsipras et al.^59^ has revealed that the annotation pipeline for ImageNet does not reflect the intention of its development for the purpose of object recognition in images. They note that ImageNet, constructed with the constraint of a single label per image, had its labels largely determined by crowdworkers indicating the visual presence of that object in the image. This has led to issues with how labels are applied, particularly to images with multiple objects, where the class of interest could include a background or obscured object that would be an unsuitable result for the image classification task of that particular photo. Furthermore, the nature of image retrieval for the annotation tasks biases the crowdworkers’ response to the labeling prompt, making them much less effective at filtering out unsuitable examples for a class category. This is just one of several inconsistencies and biases in the data that hints at larger annotation patterns that mischaracterize the real-world tasks these datasets are meant to represent, and the broader impact of data curation design choices in determining the quality of the final dataset.

Dataset documentation practices have also been a central focus, especially as dataset development processes are increasingly being recognized as a source of algorithmic unfairness. A recent study of publications that leverage Twitter data found data decisions were heavily under-specified and inconsistent across publications.^60^ Scheuerman et al.^61^ found a widespread under-specification of annotation processes relating to gender and racial categories within facial analysis datasets. Several dataset documentation and development frameworks have been proposed in an effort to address these concerns, with certain frameworks looking to not just capture characteristics of the output dataset but also report details of the procedure of dataset creation for better transparency and accountability.^62^, ^63^, ^64^, ^65^, ^66^

The lack of rigorous and standardized dataset documentation practices has contributed to reproducibility concerns. For example, recent work by Recht et al.^67^ undertook the laborious task of reconstructing ImageNet, following the original documented dataset construction process in an effort to test the generalization capabilities of ImageNet classifiers. Despite mirroring the original collection and annotation methods—including leveraging images from the same time period—the newly constructed dataset was found to have different distributional properties. The differences were largely localized to variations in constructing target labels from multiple annotations. More specifically, different thresholds for inter-annotator agreement were found to produce vastly different datasets, indicating that so-called ground truth labels in datasets do not correspond to truth.

### Summary

This section has centered on issues with dataset contents and structures, and the representational harms, spurious correlations, problem legitimization, and haphazard collection, annotation, and documentation practices that are endemic to many machine learning datasets. In the next section, we review methods which have been developed to address some of these issues.

## Dataset in(tro)spection

“For any sociotechnical system, ask, ‘what is being looked at, what good comes from seeing it, and what are we *not* able to see?’”—Mike Ananny and Kate Crawford (*Seeing without knowing: Limitations of the transparency ideal and its application to algorithmic accountability*)^68^

The massive sizes of contemporary machine learning datasets make it intractable to thoroughly scrutinize their contents,^69^ and thus it is hard to know where to begin looking for the kinds of representational and statistical biases outlined in the previous section. Indeed, a culture characterized by a desire to harness large datasets without questioning what is in them or how it got there, no matter how unsavory the details might be, produces what machine learning researcher Vinay Prabhu calls the “abattoir effect.”^70^ While many of the dysfunctional contents discovered in datasets were found by using intuition and domain expertise to construct well-designed dataset ablations and audits, some of the most disturbing were found by manually combing through the data.

Further insight into issues with dataset contents can be found in work that attempts to identify and address some of the problems outlined in the previous section. In this section, we review a variety of methods for exploring the contents of datasets in support of discovering and mitigating the issues that lurk within datasets.

### Inspection

Birhane and Prabhu,^27^ summarized in the previous section, and Pipkin^71^ show how meticulous manual audits of large datasets are compelling ways to discover the most surprising and disturbing contents therein. Pipkin spent hundreds of hours watching the entirety of MIT’s “Moments in Time” video dataset,^72^ finding shocking and unexpected footage of violence, assault, and death. They provocatively point out, in a reflection on the process of developing their artistic intervention *Lacework*, that the researchers who commission the curation of massive datasets may have less intimate familiarity with the contents of these datasets than those who are paid to look at and label individual instances, and, as we discuss in Labor, there is growing awareness of the need to better support the workers at the front lines of the often grim and under-valued work of data labeling. Caswell et al.^73^ show the value of manual audits of multilingual corpora to highlight the dubious quality of many datasets used for language model training. Their team of human volunteers, with proficiency in about 70 languages altogether, found that several corpora scraped from the web are rife with examples of mistranslated text and mislabeled linguistic content (i.e., content in a particular language labeled erroneously as belonging to another language).

### Introspection

While manual audits have provided invaluable insights into the contents of datasets, as datasets swell in size this technique is not scalable. Recent work has proposed algorithmic interventions that assist in the exploration and adjustment of datasets. Some methods leverage statistical properties of datasets to surface spurious cues and other possible issues with dataset contents. The AfLite algorithm proposed by Sakaguchi et al.^74^ provides a way to systematically identify dataset instances that are easily gamed by a model, but in ways that are not easily detected by humans. This algorithm is applied by Le Bras et al.^75^ to a variety of NLP datasets, and they find that training models on adversarially filtered data leads to better generalization to out-of-distribution data. In addition, recent work by Swayamdipta et al.^76^ proposes methods for performing exploratory data analyses based on training dynamics that reveal edge cases in the data, bringing to light labeling errors or ambiguous labels in datasets. Northcutt et al.^77^ combine an algorithmic approach with human validation to surface labeling errors in the test set for ImageNet.

Han et al.^78^ demonstrate the application of influence functions, originally introduced by Koh and Liang^79^ as a way to identify the influence of particular training examples on model predictions, to the discovery of data artifacts. The REVISE tool by Wang et al.^80^ can be used to identify unequal representation in image description datasets by leveraging features of the images and the corresponding texts. Using their tool, they spot that images of outdoor athletes are overwhelmingly labeled as men, and that in images where a person is too small for any sort of gender to be told at all, they are still labeled as men.

In response to a proliferation of challenging perturbations derived from existing datasets to improve generalization capabilities and lessen the ability for models to learn shortcuts, Liu et al.^81^ propose “inoculation by fine-tuning” as a method for interpreting what model failures on perturbed inputs reveal about weaknesses of training data (or models). Recent papers also outline methodologies for leveraging human insight in the manual construction of counterfactual examples that complement instances in NLP datasets to promote better generalization.^82^^,^^83^

The case of VQA-CP^84^ provides a cautionary tale of when a perturbed version of a dataset is, itself, prone to spurious cues. This complement to the original Visual Question Answering (VQA) dataset, consisting of VQA instances redistributed across train and test sets as an out-of-domain benchmark for the task, was found to be easy to “solve” with randomly generated answers. Cleverly designed sabotages that are meant to strengthen models’ ability to generalize may ultimately follow the same patterns as the original data, and are thus prone to the same kinds of artifacts. While this has prompted attempts to make models more robust to any kind of dataset artifact, it also suggests that there is a broader view to be taken with respect to rethinking how we construct datasets for tasks overall.

Considering that datasets will always be imperfect representations of real-world capabilities, recent work proposes methods of mitigating the impacts of noise in data on model performance. Teney et al.^85^ propose an auxiliary training objective using counterfactually labeled data to guide models toward better decision boundaries. He et al.^86^ propose the DRiFT algorithm for “unlearning” dataset bias. Sometimes, noise in datasets is not symptomatic of statistical anomalies or labeling errors, but rather, a reflection of variability in human judgment. Pavlick and Kwiatkowski^87^ find that human judgment on natural language inference tasks is variable, and that machine evaluation on this task should reflect this variability.

Many of the methods outlined in this section crucially rely on statistical patterns in the data to surface problematic instances; it is up to human judgment to make sense of the nature of these problematic instances, whether they represent logical inconsistencies with the task at hand, cases of injustice, or both. In addition, while a variety of recent papers have proposed methods for removing spurious cues from training data or “de-biasing” models, recent work has shown that this can be damaging for model accuracy.^88^

In contrast to a focus on statistical properties of datasets as a site for addressing and mitigating harms, Denton et al.^89^ propose a research agenda in the “data genealogy” paradigm that promotes critical assessment of the design choices with respect to the data sources, theoretical motivations, and methods used for constructing datasets. Prospective accounting for dataset contents using some of the methods discussed at the end of the previous section can offset the potential of post-hoc documentation debt that can be incurred otherwise.^69^

### Summary

In this section we have reviewed a variety of works that address dataset content issues by providing lenses on data for inspection and introspection. We emphasize that procedural dataset modifications and bias mitigation techniques are only useful insofar as the dataset in question itself represents a well-designed task. In making lemonade from lemons, we must ensure the lemons are not ill-gotten or poorly formed.

## Dataset culture

“Every data set involving people implies subjects and objects, those who collect and those who make up the collected. It is imperative to remember that on both sides we have human beings.”—Mimi Ọnụọha (*The Point of Collection*)^90^

A final layer of critiques looks at the culture around dataset use in machine learning. In this section, we examine how common practices in dataset usage impact society at large by reviewing papers that ask: What are issues with the broader culture of dataset use? How do our dataset usage, storage, and re-usage practices wrench data away from their contexts of creation? What are the labor conditions under which large-scale crowd-sourced datasets are produced? Finally, what can be learned from looking at machine learning dataset culture from a legal perspective?

### Benchmarking practices

Benchmark datasets play a critical role in orienting the goals of machine learning communities and tracking progress within the field.^5^^,^^89^ Yet, the near singular focus on improving benchmark metrics has been critiqued from a variety of perspectives. Indeed, the current benchmarking culture has been criticized as having the potential to stunt the development of new ideas.^91^ NLP researchers have exhibited growing concern with the singular focus on benchmark metrics, with several calls to include more comprehensive evaluations—including reports of energy consumption, model size, fairness metrics, and more—in addition to standard top-line metrics.^92^, ^93^, ^94^ Sculley et al.^95^ examine the incentive structures that encourage singular focus on benchmark metrics—often at the expense of empirical rigor—and offer a range of suggestions including incentivizing detailed empirical evaluations, including negative results, and sharing additional experimental details. From a fairness perspective, researchers have called for the inclusion of disaggregated evaluation metrics, in addition to standard top-line metrics, when reporting and documenting model performance.^96^

The excitement surrounding leaderboards and challenges can also give rise to a misconstrual of what high performance on a benchmark actually entails. In response to the recent onslaught of publications misrepresenting the capabilities of BERT language models, Bender and Koller^97^ encourage NLP researchers to be attentive to the limitations of tasks and include error analysis in addition to standard performance metrics.

Sen et al.^58^ have questioned the legitimacy of the notion of a gold standard dataset for certain tasks, empirically demonstrating divergences between gold standards set by AMT workers and those from other communities. Other data-oriented fields have grappled with the politics inherent in quantification and measurement practices.^98^, ^99^, ^100^ Jacobs and Wallach^101^ locate dataset measurement concerns as a key factor underlying unfair outcomes of algorithmic systems and propose that machine learning practitioners adopt measurement modeling frameworks from the quantitative social sciences.

### Data management and distribution

Secure storage and appropriate dissemination of human-derived data is a key component of data ethics.^102^ To have a culture of care for the subjects of the datasets we make use of requires us to prioritize the well-being of the subjects in the dataset throughout collection, development, *and* distribution. To do so systematically, the machine learning community still has much to learn from other disciplines with respect to how they handle the data of human subjects. Unlike in the social sciences or medicine, the machine learning field has yet to develop the data management practices required to store and transmit sensitive human data.

Metcalf and Crawford^103^ go so far as to suggest the re-framing of data science as human subjects research, indicating the need for institutional review boards and informed consent as researchers make decisions about other people’s personal information. Particularly in consideration of an international context, where privacy concerns may be less regulated in certain regions, the potential for data exploitation is a real threat to the safety and well-being of data subjects.^104^ As a result, those that are the most vulnerable are at risk of losing control of the way in which their own personal information is handled. Without individual control of personal information, anyone who happens to be given the opportunity to access their unprotected data can act with little oversight, potentially against the interests or well-being of data subjects. This can become especially problematic and dangerous in the most sensitive contexts, such as personal finance information, medical data, or biometrics.^105^

However, machine learning researchers developing such datasets rarely pay attention to this necessary consideration. Researchers will regularly distribute biometric information—for example, face image data—without so much as a distribution request form or required privacy policy in place. Furthermore, the images are often collected without any level of informed consent or participation.^6^^,^^50^^,^^106^ In the context of massive data collection projects, the potential harms extend beyond those that can be addressed with individual consent. Solove^107^ provides a thoughtful overview of privacy as both a societal and individual value.

Even when these datasets are flagged for removal by the creators, researchers will still attempt to make use of that now illicit information through derivative versions and backchannels. For example, Peng^108^ finds that, after certain problematic face datasets were removed, hundreds of researchers continued to cite and make use of copies of this dataset months later. Without any centralized structure of data governance for the research in the field, it becomes nearly impossible to take any kind of significant action to block or otherwise prevent the active dissemination of such harmful datasets.

Security concerns arise due to the manner in which large-scale datasets are curated and disseminated through a web-scraping paradigm. For example, it was recently discovered that one of the URLs in the ImageNet dataset that originally pointed to an image of a bat instead linked to malware, potentially making dataset users vulnerable to hacking.^109^ Carlini et al.^110^ also illustrate how large language models can be prodded to disgorge sensitive, personally identifying information they have picked up from their training data.

Best practices for sharing and managing datasets are a burgeoning area of research in NLP. In addition to a comprehensive accounting for the motivations and contents of abusive language datasets, Vidgen and Derczynski^111^ provide several suggestions for the responsible dissemination of such data, including the establishment of data trusts, platform-supported datasets, and the use of synthetic data.

### Use and reuse

Several scholars have written on the importance of reusable data and code for reproducibility and replicability in machine learning,^112^^,^^113^ and the publication of scientific data is often seen as an unmitigated good, either in the pursuit of reproducibility^114^ or as a means of focusing research effort and growing research communities (e.g., through shared task evaluations^115^). Here, we want to consider the potential pitfalls of taking data that had been collected for one purpose and using it for one in which it was not intended, particularly when this data reuse is morally and ethically objectionable to the original curators. Science and technology scholars have considered the potential incompatibilities and reconstructions needed in using data from one domain in another.^116^ Indeed, Strasser and Edwards discuss several major questions for Big Data in science and engineering, asking critically “Who owns the data?” and “Who uses the data?”.^117^ Although in Legal perspectives we discuss ownership in a legal sense, ownership also suggests an inquiry into who the data have come from, such as the “literal […] DNA sequences” of individuals^117^ or other biometric information. In this case, considering data reuse becomes a pivotal issue of benchmark datasets.

Instances of data reuse in benchmarks are often seen in the scraping and mining context, especially when it comes to Flickr, Wikipedia, and other openly licensed data instances. Many of the instances in which machine learning datasets drawn from these and other sources in ways that incur serious privacy violations are well-documented by Harvey and LaPlace,^106^ who discuss instances of scraping Flickr and other image hosting services for human images without explicit user consent.

The reuse of data can involve reusing data from one context and using this decontextualized data for machine learning applications. This dynamic is exemplified well by historian of science Joanna Radin’s exploration of the peculiar history of the Pima Indians Diabetes Dataset (PIDD) and its introduction into the UCI Machine Learning Repository.^118^ The PIDD has been used thousands of times as a “toy” classification task and currently lives in the UCI repository, a major repository for machine learning datasets. The data were collected by the National Institutes of Health from the Indigenous community living at the Gila River Indian Community Reservation, which had been extensively studied and restudied for their high prevalence of diabetes. In her history of this dataset, Radin is attentive to the politics of the creation and processing of the data itself. The fact that “data was used to refine algorithms that had nothing to do with diabetes or even to do with bodies, is exemplary of the history of Big Data writ large”. Moreover, the residents of the Reservation, who refer to themselves as the Akimel O’odham, had been the subject of intense anthropological and biomedical research, especially due to a high prevalence of diabetes, which in and of itself stemmed from a history of displacement and settler-colonialism. However, their participation in research had not yielded any significant decreases in obesity or diabetes among community members.

Another concerning example of data reuse occurs when derivative versions of an original dataset are distributed—beyond the control of its curators—without any actionable recourse for removal. The DukeMTMC (Duke Multi-Target, Multi-Camera) dataset was collected from surveillance video footage from eight cameras on the Duke campus in 2014, used without consent of the individuals in the images and distributed openly to researchers in the US, Europe, and China. After reporting in the *Financial Times*^119^ and research by Harvey and LaPlace,^106^ the dataset was taken down on June 2, 2019. However, Peng^108^ has recently highlighted how the dataset and its derivatives are still freely available for download and used in scientific publications. It is nearly impossible for researchers to maintain control of datasets once they are released openly or if they are not closely supervised by institutional data repositories.

Across all of these instances of data use and reuse we observe that, when datasets are not created specifically and only for the use of machine learning research, there is the potential for a culture clash between the data practices of machine learning and the data practices of the field where the data comes from. Currently, machine learning (and computer science more generally) is relatively powerful compared with many other academic disciplines and risks exporting its data practices, whereas we argue that, as a field, we should be looking to learn from other fields’ approaches to appropriate and situated data handling (see, e.g., Jo and Gebru^47^). We further note that a culture change around data use and reuse is a field-level problem that will require community buy-in and field-level allocation of resources to address. For example, to address the ways in which deprecated datasets, such as DukeMTMC, continue to circulate it is not enough to create a central repository that holds information about dataset retraction and other updates; researchers must also be incentivized and trained to consult such repositories.

### Labor

As the machine learning community has increasingly turned to the cheap and scalable work forces offered by crowd sourcing platforms, there has been growing concern regarding the working conditions of those laboring on machine learning datasets. Data annotation is often cast as unskilled work—work *anyone* can perform—which in turn contributes to a dehumanizing and alienating work experience. For example, Irani^120^ describes how crowdwork platforms, such as AMT, create a hierarchy of data labor, positioning crowdwork as menial work relative to the innovative work of those leading dataset development. Miceli et al.^55^ discuss how, in commercial data annotation companies, power asymmetries and company hierarchies affect the work output of data annotation teams. Framing data annotation as unskilled work frames crowdworkers as essentially interchangeable, and creates the infrastructural conditions of precarity and invisibility.^121^, ^122^, ^123^ For example, crowd-sourced data annotation is typically mediated through digital interfaces that distance the crowdworkers from the dataset developers constructing annotation tasks, rendering both the workers and the labor concerns they might face invisible.^124^^,^^125^ Such labor concerns include low and unstable wages, unfair treatment by task requesters, and barriers to worker solidarity and collective action.^126^, ^127^, ^128^

In response to these growing concerns, guidelines and tools for task creators have been developed to help facilitate fair pay^129^^,^^130^ and interventions oriented at crowdworkers directly have been developed to support worker solidarity^124^^,^^131^ and fair pay.^132^ Gray and Suri^128^ also discuss corporate interventions, such as providing collaborative online discussion spaces, offline shared workspaces, and portable reputation systems, as well as governmental responses, such as the construction of worker guilds, unions, and platform cooperatives, and the provision of a social safety network for these precarious workers.

As personal data are increasingly commodified by technology companies and harvested at scale to improve proprietary machine learning systems, often in ways that are by turns inscrutable or distasteful to the general public,^133^ recent proposals call for not only re-framing data as labor,^134^ but also for “data strikes” in which users collectively withhold their data as a means to shift the power imbalance back toward subjects who are not compensated for the ambient collection of their data.^135^

### Legal perspectives

The above subsections surveyed a range of literature critiquing different aspects of dataset culture in machine learning. In this section, we review literature that looks at the collection and use of datasets from a legal perspective, considering both the legal risks that dataset collectors incur and the extent to which existing legal frameworks protect data subjects. Benchmark datasets are often mined from the internet, collecting data instances that have various levels of licensing attached and storing them into a single repository. Different legal issues arise at each stage in the data-processing pipeline, from collection to annotation, from training to evaluation, from inference and the reuse of downstream representations, such as word embeddings and convolutional features.^136^ Legal issues also arise that impact a host of different people in the process, including dataset curators, AI researchers, copyright holders, data subjects (those people whose likenesses, utterances, or representations are in the data), and consumers (those who are not in the data but are impacted by the inferences of the AI system). Different areas of law can protect (and also possibly harm) each of the different actors in turn.^137^

Benchmark datasets are drawn from a number of different sources, each with a different configuration of copyright holders and permissions for their use in training and evaluation in machine learning models. For instance, ImageNet was collected through several image search engines where licensing/copyright restrictions on data instances in those images are unknown.^138^ The ImageNet project does not host the images on their website, and therefore sidesteps the copyright question by claiming that they operate like a search engine^139^ (fn. 36). PASCAL VOC was collected via the Flickr API, meaning that the images were all held through the Creative Commons license.^140^ Open licenses, such as Creative Commons, allow for training of machine learning models under fair use doctrine.^141^ Faces in the Wild and Labeled Faces in the Wild were collected through Yahoo News, and via an investigation of the captions on the images we can see that the major copyright holders of those images are news wire services, including the Associated Press and Reuters.^142^ Other datasets are collected in a studio environment, where images were taken by dataset curators and therefore are copyright holders, which avoids potential copyright issues.

US copyright law is not well-suited to cover the range of uses of benchmark datasets, and there is limited case law establishing precedent in this area. Legal scholars have defended the use of copyrighted material for data science and machine learning by suggesting that this material’s usage is protected by fair use, since it entails the non-expressive use of expressive materials.^143^ In contrast, Levendowski^139^ has argued that copyright is actually a useful tool for battling algorithmic bias by offering a larger pool of works from which machine learning practitioners can draw from. She argues that, given that pre-trained representations, such as word2vec and other word embeddings, suffer from gender and racial bias,^144^^,^^145^ and other public domain datasets are older or obtained through means likely to result in amplified representation of stereotypes and other biases in the data (e.g., the Enron text dataset), that using copyrighted data can battle biased datasets and their use would fall under copyright’s fair use exception.

Even in cases in which all data were collected legally from a copyright perspective—such as through open licenses, like Creative Commons—many downstream questions remain, including issues about privacy, informed consent, and procedures of opt-out.^141^ O’Sullivan^109^ discusses how technically legal uses of personal data that are not anticipated by or fully disclosed to the original owners of the data, e.g., the use of images scraped from the web to train facial recognition algorithms, constitute the ethical equivalent of data theft. Copyright guarantees are not sufficient protections for safeguarding privacy rights of individuals, as seen in the collection of images for the Diversity in Faces and MegaFace datasets.^50^^,^^119^ Potential privacy violations arise when datasets contain biometric information that can be used to identify individuals, including faces, fingerprints, gait, and voice among others. However, at least in the US, there is no national-level privacy law that deals with biometric privacy. A patchwork of laws exist in Illinois, California, and Virginia that have the potential to safeguard the privacy of data subjects and consumers. However, only the Illinois Biometric Privacy law requires corporate entities to provide notice to data subjects and obtain their written consent.^137^

The machine learning and AI research communities have responded to this crisis by attempting to outline alternatives to licensing which make sense for research and benchmarking practices more broadly. The Montreal Data License (https://montrealdatalicense.com/) outlines different contingencies for a particular dataset, including whether the dataset will be used in commercial versus non-commercial settings, whether representations will be generated from the dataset, whether users can annotate the label or use subsets of it, and more.^136^ This is a step forward in clarifying the different ways in which the dataset can be used once it has been collected, and therefore is a clear boon for AI researchers who create their own data instances, such as photos developed in a studio or text or captions written by crowdworkers. However, this does not deal with the larger issue of the copyright status of data instances scraped from the web, or the privacy implications of those data instances.

### Summary

In this section, we have shed light on issues around benchmarking practices, dataset use and reuse, and the legal status of benchmark datasets. These issues are more about the peculiar practices of data in machine learning culture, rather than the technical challenges associated with benchmark datasets. In this way, we want to highlight how datasets work *as* culture—that is, “not [as] singular technical objects that enter into many different cultural interactions, but … rather [as] unstable objects, culturally enacted by the practices people use to engage with them”.^146^ Interrogating benchmark datasets from this view requires us to expand our frame from simply technical aspects of the system, to thinking how datasets intersect with communities of practice, communities of data subjects, and legal institutions.^147^

## Conclusion

“Not all speed is movement.”—Toni Cade Bambara (*On the Issue of Roles*)^148^

In this paper, we present a survey of issues in dataset design and development, as well as reflections on the current broader culture of dataset use in machine learning. A viewpoint internal to this culture values rapid and massive progress: ever larger training datasets, used to train ever larger models, which post ever higher scores on ever harder benchmark tasks developed at a quicker and quicker pace. What emerges from the papers we survey, however, is a viewpoint, largely external to the current culture of dataset use, which reveals intertwined scientific and ethical concerns appealing to a more careful, systems-level and detail-oriented strategy.

The critiques of dataset design and development we survey in this paper highlight various different kinds of pitfalls: first, there are challenges with representation wherein datasets are biased both in terms of which data subjects are predominantly included and whose gaze is represented. Second, we find issues with the artifacts in the data, which machine learning models can easily leverage to “game” the tasks. Third, we find evidence of whole tasks which are spurious, where success is only possible given artifacts because the tasks themselves do not correspond to reasonable real-world correlations or capabilities. Finally, we find critiques of insufficiently careful data annotation and documentation practice, which erode the foundations of any scientific inquiry based on these datasets.

A variety of methods have been applied to examining dataset contents to surface some of the quality issues and harmful contents within. Attempts to rehabilitate datasets or models starting from the flawed datasets themselves further reinforce the problems outlined in the critiques of dataset design and development. The development of adversarial datasets or challenge sets, while possibly removing some spurious cues, does not address most of the other issues with either the original datasets or the research paradigm.

Critiques of the dataset culture itself focus on the overemphasis on benchmarking to the exclusion of other evaluation practices, legal and ethical issues in data management, distribution, and reuse, and labor practices in data curation. Hyper-focus on benchmarking pushes out work that connects models more carefully to their modeling domain and approaches not optimized for the available crop of benchmarks. The papers we surveyed suggest a need for work that takes a broader view than is afforded by the one-dimensional comparison of systems typical of benchmarks. Furthermore, critiques of data management and distribution show the need for growing a culture of care for the subjects of datasets in machine learning, i.e., to keep in mind that “data are people” and behave appropriately toward the people from whom we collect data.^149^ Reflections on issues of data reuse emphasize the connection between data and its context, and the risks of harm (to data subjects and others) that arise when data is disconnected from its context and carried to and recontextualized in new domains. Legal vulnerabilities inherent to current data collection and distribution practices in machine learning as well as the often precarious and under-compensated nature of dataset work reveal the complexities of data development and use within the context of society. Overall, these critiques shed light on the need for the kind of broader systems-level thinking required to navigate an under-valued although clearly necessary aspect of machine learning development.

What paths forward are visible from this broader viewpoint? We argue that fixes that focus narrowly on improving datasets by making them more representative or more challenging might miss the more general point raised by these critiques—namely that data challenges are in general under-considered in the field. If such data issues are left unaddressed, the field will be trapped in the Sisyphean task of finding and fixing dataset flaws rather than taking the necessary step back to address the more systematic issues at play. This renewed focus is essential to making progress as a field, so long as notions of progress are largely defined by performance on datasets. At the same time, we wish to recognize and honor the liberatory potential of datasets, when carefully designed, to make visible patterns of injustice in the world such that they may be addressed (see, for example, the work of Data for Black Lives [https://d4bl.org/]). Recent work by Register and Ko^150^ illustrates how educational interventions that guide students through the process of collecting their own personal data and running it through machine learning pipelines can equip them with skills and technical literacy toward self-advocacy—a promising lesson for the next generation of machine learning practitioners and for those impacted by machine learning systems. We also recognize the need for a fundamental shift in the incentive structures that guide how machine learning practitioners prioritize dataset-related tasks. The introduction of a “Datasets and Benchmarks Track”^151^ at the Neural Information Processing Systems Conference 2021, which will incentivize data-focused research, indicates a positive step in this direction.

We hope the response to this work goes beyond optimizing datasets to be “bigger” and “better”—a goal that does nothing to challenge the current paradigm of techniques idolizing speed and scale. Instead, we aspire for this survey to also prompt a more cautious and complex view of the considerations involved with data in the machine learning field. We advocate for a turn in the culture toward carefully collected datasets that are rooted in their original contexts, distributed in ways that respect the intellectual property and privacy rights of data creators and data subjects, and constructed in conversation with impacted stakeholders or domain experts. This is how we hope to arrive at datasets that faithfully embody tasks targeting realistic capabilities and that acknowledge the humanity of those represented within the data, in addition to those participating in the process of its creation. Such datasets will undoubtedly be more expensive to create, in time, money, and effort, but this is small price to pay for the consideration of the human lives at stake.
